# Exploring the Olfactory Recognition of *Elaeagnus angustifolia* Volatiles in *Anoplophora glabripennis* Through Antennal Transcriptome Analysis and Molecular Characterization of Classic OBPs

**DOI:** 10.3390/insects17070666

**Published:** 2026-06-25

**Authors:** Lubing Wang, Chunchun Li, Suqin Shang, Zhuandi Pei, Youssef Dewer, Lixiang Wang

**Affiliations:** 1 Biocontrol Engineering Laboratory of Crop Diseases and Pests of Gansu Province, College of Plant Protection, Gansu Agricultural University, Lanzhou 730070, China; wanglubing@gsau.edu.cn (L.W.); lcc@gsau.edu.cn (C.L.); shangsq@gsau.edu.cn (S.S.); 1073324020458@st.gsau.edu.cn (Z.P.); 2 Phytotoxicity Research Department, Central Agricultural Pesticide Laboratory, Agricultural Research Center, Dokki, Cairo 12618, Egypt; dewer72@yahoo.com

**Keywords:** *Anoplophora glabripennis*, *Elaeagnus angustifolia*, odorant-binding protein

## Abstract

*Anoplophora glabripennis* is a destructive forest pest. *Elaeagnus angustifolia* attracts *A. glabripennis* for feeding and oviposition, but its gum encapsulates and kills the eggs, functioning as a dead-end trap tree. However, the olfactory mechanisms by which *A. glabripennis* recognizes *E. angustifolia* volatiles remain unclear. In this study, we analyzed the antennal transcriptome of female adult *A. glabripennis* exposed to *E. angustifolia* volatiles. Ten OBP genes were significantly up-regulated in response to the volatiles, including six Classic OBPs and four Minus-C OBPs (log_2_ fold changes: 1.02–3.01). qRT-PCR showed *AglaOBP1*/*2*/*3* were highly and specifically expressed in the antennae, suggesting key olfactory roles. Static molecular docking showed that all three OBPs bound 22 *E. angustifolia* volatiles, each displaying the highest affinity for (+)-Longifolene, with AglaOBP1 exhibiting the strongest binding. Nevertheless, 200 ns MD simulations revealed a shift: the AglaOBP3–(+)-Longifolene complex displayed the greatest structural stability, not AglaOBP1. MM/PBSA corrected the initial docking screen and confirmed that AglaOBP3 had the strongest thermodynamic binding affinity for (+)-Longifolene (ΔG_bind_ = −30.94 ± 2.57 kcal·mol^−1^). This study provides novel molecular insights into the olfactory recognition of *E. angustifolia* volatiles in *A. glabripennis*, laying a foundation for future functional validation and sustainable pest management.

## Simple Summary

The Asian longhorned beetle (*Anoplophora glabripennis*) is a highly destructive wood-boring pest that has spread to over 20 countries, causing severe damage to forest ecosystems in affected regions. Russian olive (*Elaeagnus angustifolia*) has a natural “dead-end trap” against this pest: it attracts *A. glabripennis* to feed and lay eggs but then produces a sticky gum that traps and kills the eggs before they can hatch. We found that three specific proteins in *A. glabripennis* antennae likely play key roles as “scent detectors” in recognizing the smell of *E. angustifolia*. These proteins can bind to many different scent molecules released by *E. angustifolia*, and one key molecule shows a high binding affinity. These findings provide new molecular insights into the olfactory basis of this natural trap-tree system and offer candidate targets for future functional tests and eco-friendly pest control.

## 1. Introduction

Insects rely on highly refined olfactory systems to navigate complex ecological environments, enabling them to locate hosts, identify mates, select oviposition sites, and avoid threats. Plant-derived volatile organic compounds (VOCs) are central mediators of these interactions, serving as critical chemical cues that shape insect behavior [1,2]. Odor detection occurs primarily in the antennae, which house specialized sensilla containing olfactory sensory neurons. Within these sensilla, soluble olfactory proteins facilitate the initial molecular events of odor recognition [3].

Among these proteins, odorant-binding proteins (OBPs) constitute the first molecular interface between environmental odorants and neuronal receptors. OBPs bind hydrophobic volatile molecules and transport them through the sensillum lymph to odorant receptors (ORs), initiating signal transduction and downstream behavioral responses [1,4,5]. Based on conserved cysteine motifs, OBPs are classified into Classic, Plus-C, Minus-C, Dimer, and Atypical subfamilies [6,7]. Although numerous OBPs have been identified across insect taxa, the functional specificity of many members—particularly in *A. glabripennis* detection of *E. angustifolia* volatiles—remains poorly understood.

The Asian longhorned beetle (*Anoplophora glabripennis*) is a globally invasive wood-boring pest and a regulated quarantine species. Native to East Asia, it has spread to over 20 countries via international trade in solid wood packing materials [8]. The beetle infests a broad range of hardwood hosts, including Populus, Salix, Ulmus, Acer, and Betula, causing extensive ecological and economic damage. Larval tunneling disrupts vascular tissues, while adult feeding further weakens host trees [9,10,11,12]. Its concealed larval development and prolonged life cycle complicate control efforts, and current management strategies remain insufficient for long-term suppression [13,14].

*Elaeagnus angustifolia* (Russian olive) represents a unique ecological paradox. Although it emits volatiles that strongly attract adult *A. glabripennis* for feeding and oviposition, it subsequently secretes gum at oviposition sites, encapsulating and killing deposited eggs. This rare “dead-end trap” interaction has been validated through systematic field investigations in northwestern China [15,16,17]. Plant volatile perception is therefore a prerequisite for the lure–kill mechanism, positioning olfactory recognition as a critical molecular determinant of this interaction [18].

OBPs are key mediators of plant volatile recognition. In several insect species, Classic OBPs have been shown to directly regulate plant selection behavior through selective ligand binding [19,20]. In *A. glabripennis*, transcriptomic studies have identified multiple OBP genes [21,22]; however, their functional roles—particularly those of Classic OBPs—in the perception of *E. angustifolia* volatiles remain largely unexplored. This gap limits both mechanistic understanding and the rational optimization of the *E. angustifolia*-based trap-tree system.

In this study, we constructed the antennal transcriptome of newly emerged female *A. glabripennis* following a 6 h exposure to volatiles from *E. angustifolia* seedlings under no-food and no-water conditions, with unexposed insects as controls. Differentially expressed OBPs were identified, with emphasis on Classic OBPs for phylogenetic characterization and tissue-specific expression analysis. We further employed molecular docking, molecular dynamics simulations, and MM/PBSA free energy calculations to elucidate the structural basis of OBP–ligand interactions. By integrating transcriptomic profiling with computational structural biology, this study advances mechanistic understanding of *E. angustifolia* volatile recognition in the trap-tree system and provides key molecular clues for the future functional verification of Classic OBPs in *A. glabripennis*.

## 2. Materials and Methods

### 2.1. Insect Rearing and Tissue Dissection

*A. glabripennis* larvae were collected in May 2024 from heavily infested *Populus gansuensis* trees in the Hexi Corridor, Gansu Province, China. After felling, infested logs were cut into 1 m sections, and both ends were sealed with paraffin to reduce moisture loss. Logs were transported to the laboratory and maintained at 25 ± 1 °C until adult emergence.

Newly emerged female adults were transferred to a rearing cage (2 m × 2 m × 2 m) containing three-year-old *E. angustifolia* seedlings. Seedlings were enclosed with a mesh net to facilitate direct exposure to plant volatiles. For the control group, newly emerged female adults were placed into an identical empty rearing cage. The control cage was placed in a separate laboratory room and maintained under completely matched environmental conditions (25 ± 1 °C, consistent photoperiod and humidity) as the treatment cage. No food or water was provided to either group during the 6 h experimental period. After 6 h of parallel rearing, antennae were dissected from females in both groups using sterile scissors. All dissected antennae were immediately immersed in RNAlater solution (Invitrogen, Carlsbad, CA, USA), flash-frozen in liquid nitrogen, and stored at −80 °C until RNA extraction.

### 2.2. RNA Extraction and Transcriptome Sequencing

Total RNA was extracted using RNAiso Plus reagent (TaKaRa, Shiga, Japan) following the manufacturer’s protocol. Each biological replicate consisted of three pairs of antennae pooled together, with three independent replicates per group (Control1–Control3 and Treat1–Treat3). RNA integrity and concentration were evaluated using an Agilent 2100 Bioanalyzer (Agilent Technologies, Santa Clara, CA, USA). High-quality RNA samples were used for cDNA library construction and sequenced on the Illumina NovaSeq X Plus platform (Guangzhou Genedenovo Biotechnology Co., Ltd., Guangzhou, China).

### 2.3. Transcript Reconstruction and Functional Annotation

Raw reads were filtered using fastp v0.18.0 to remove adapter sequences, reads containing more than 10% ambiguous nucleotides (N), and low-quality reads with more than 50% of bases having a Q-value ≤ 20 to obtain high-quality clean reads. Clean reads were aligned to the ribosomal RNA (rRNA) database using Bowtie2 v2.2.8, and rRNA-mapped reads were removed. An index of the reference genome (NCBI Assembly ID: GCA_000390285.2) was constructed, and the remaining clean reads were aligned to the reference genome using HISAT2 v2.1.0 with default parameters, followed by transcript reconstruction using StringTie v1.3.1. Gene expression levels were quantified using the RSEM (0.5.1) package. Expression abundance was calculated as transcripts per million (TPM). Subsequently, differentially expressed genes (DEGs) between the treatment and control groups were identified using DESeq2, with thresholds of false discovery rate (FDR) < 0.05 and |log_2_FC| > 1.

For functional annotation of all genes, we directly adopted the pre-existing annotations of the reference genome covering Gene Ontology (GO), Kyoto Encyclopedia of Genes and Genomes (KEGG), euKaryotic Orthologous Groups (KOG), Swiss-Prot, Pfam and NCBI Non-Redundant (NR) databases. Furthermore, GO and KEGG pathway enrichment analyses for DEGs were performed using the hypergeometric test implemented in the R package (3.10.1) clusterProfiler, where raw *p*-values were corrected for multiple testing using the FDR method, and terms with FDR ≤ 0.05 were defined as significantly enriched.

### 2.4. Identification and Bioinformatic Analysis of OBPs

OBP-encoding genes that were significantly upregulated in response to *E. angustifolia* volatiles were identified from the transcriptome. Candidate sequences were manually validated by BLASTx searches against the NR database using an E-value threshold of <1 × 10^−5^. Open reading frames (ORFs) were predicted using the NCBI ORF Finder, and signal peptides were identified with SignalP 6.0.

Multiple sequence alignments were performed using ClustalW and manually curated in JalView 2.11 to classic OBP subfamilies. OBP sequences from other insect species were retrieved from NCBI for comparative analysis. Phylogenetic relationships were inferred using the maximum likelihood (ML) method implemented in MEGA v12.0 with default parameters. Nodal support was assessed using 1000 bootstrap replicates. Phylogenetic trees were visualized using the Interactive Tree of Life (iTOL) platform.

### 2.5. Expression Profiling by Quantitative Real-Time PCR (qRT-PCR)

Expression patterns of identified OBP genes were evaluated by qRT-PCR across different tissues. Antennae, maxillary palps, and legs were dissected from three female and three male adults. Tissues from individuals of the same sex were pooled to form one biological replicate per tissue type. Total RNA was extracted using RNAiso Plus reagent, and first-strand cDNA was synthesized using the PrimeScript RT Reagent Kit with gDNA Eraser (TaKaRa). qRT-PCR reactions (20 μL) contained 10 μL TB Green Premix Ex Taq II, 0.8 μL of each primer, 2 μL cDNA template, and 6.4 μL RNase-free water. Thermal cycling conditions were as follows: 95 °C for 30 s; 40 cycles of 95 °C for 5 s and 60 °C for 30 s. A melt curve analysis (65–95 °C, with a 0.5 °C increment every 5 s) was performed to verify amplification specificity. RNase-free water served as the no-template control.

Gene-specific primers were designed using Primer3 (NCBI), and β-actin was used as the reference gene (Table S1). Maxillary palps from male adults were used as the calibrator group. Three biological replicates were performed, each with three technical replicates. Relative gene expression levels were calculated using the 2^−ΔΔCt^ method. Statistical significance among tissues was determined by one-way ANOVA followed by Duncan’s multiple comparison test. Statistical analyses were performed using SPSS Statistics v27, and figures were generated with Origin 2025. In addition to tissue-specific expression analysis, qRT-PCR was also performed to validate the transcriptome results using female antennae from the control and *E. angustifolia* volatile-exposed groups (the same samples used for RNA-seq). The reaction system and cycling conditions were identical to those described above.

### 2.6. Homology Modeling and Molecular Docking

Three-dimensional (3D) structures of the highly expressed OBPs that were identified via qRT-PCR from *A. glabripennis* antennae were predicted using the SWISS-MODEL online platform (https://swissmodel.expasy.org (accessed on 5 September 2025)). Templates were selected based on >30% sequence identity and >80% coverage relative to the target OBPs. Model quality was assessed using Procheck, ERRAT, and ProSA-web.

Structures of 22 *Elaeagnus angustifolia* volatiles, previously identified by our group, were retrieved from the PubChem database (https://pubchem.ncbi.nlm.nih.gov/ (19 September 2025)). Protein and ligand preprocessing included hydrogen addition, charge assignment, and definition of docking grids based on predicted binding pockets from the NCBI Conserved Domain Database and InterPro. AutoDockTools v1.5.7 was used for ligand and receptor preparation, and docking was performed using AutoDock Vina-GPU 2.1 with 150 independent runs per complex. The lowest-energy conformations were retained for further analysis. Protein–ligand interactions, including hydrogen bonding and hydrophobic contacts, were visualized using BIOVIA Discovery Studio 2021 and PyMol 3.1.

### 2.7. Molecular Dynamics (MD) Simulation

To evaluate the stability and dynamic behavior of the optimal protein–ligand complexes, 200 ns MD simulations were conducted using GROMACS 2025.3. Protein parameters were assigned with the AMBER99SB-ILDN force field, and ligands were parameterized using the GAFF force field in AmberTools 24. Partial charges for ligands were generated with ACPYPE. Each complex was solvated in a TIP3P water box, neutralized with Na^+^/Cl^−^ ions, and energy-minimized using the steepest descent algorithm (maximum force < 500 kJ/mol/nm).

Equilibration was performed under NVT and NPT ensembles for 100 ps each, using the velocity–Verlet integrator, V-rescale temperature coupling, and Parrinello–Rahman pressure coupling. Production simulations were run for 200 ns under NPT conditions with a 2 fs time step, saving trajectories every 10 ps (20,000 frames total). System temperature and pressure were maintained at 300 K and 1 bar, respectively, with constraints applied only to bonds involving hydrogen atoms.

### 2.8. Molecular Mechanics/Poisson–Boltzmann Surface Area (MM/PBSA)

Binding free energies (ΔG_bind_) of protein–ligand complexes were calculated from equilibrated MD trajectories using the MM/PBSA method. Per-residue energy decomposition was performed to identify amino acids contributing most significantly to ligand binding. Total binding energy was partitioned into van der Waals (ΔG_vdw_), electrostatic (ΔG_ele_), polar solvation (ΔG_PB_), and nonpolar solvation (ΔG_SA_) components, providing mechanistic insights into the interactions governing OBP–volatile recognition.

## 3. Results

### 3.1. Antenna Transcriptome Sequencing of A. glabripennis

cDNA libraries were constructed from the antennae of female *A. glabripennis* from control and *E. angustifolia*-exposed groups, with three biological replicates each, and sequenced on the Illumina NovaSeq X Plus platform. The mean raw read count of control samples was 52,020,159, and that of treatment samples was 47,902,960. After stringent quality filtering, control samples retained >45,093,200 clean reads, with Q20 ≥ 99.22% and Q30 ≥ 96.43%. Adapter contamination was ≤0.05%, and GC content ranged from 41.19% to 43.53%. Treatment samples showed similar metrics, confirming high-quality sequencing suitable for downstream analyses (Table S2). Raw data are available in the NCBI SRA database under accession number PRJNA1403806.

### 3.2. DEGs

**Figure 1:**
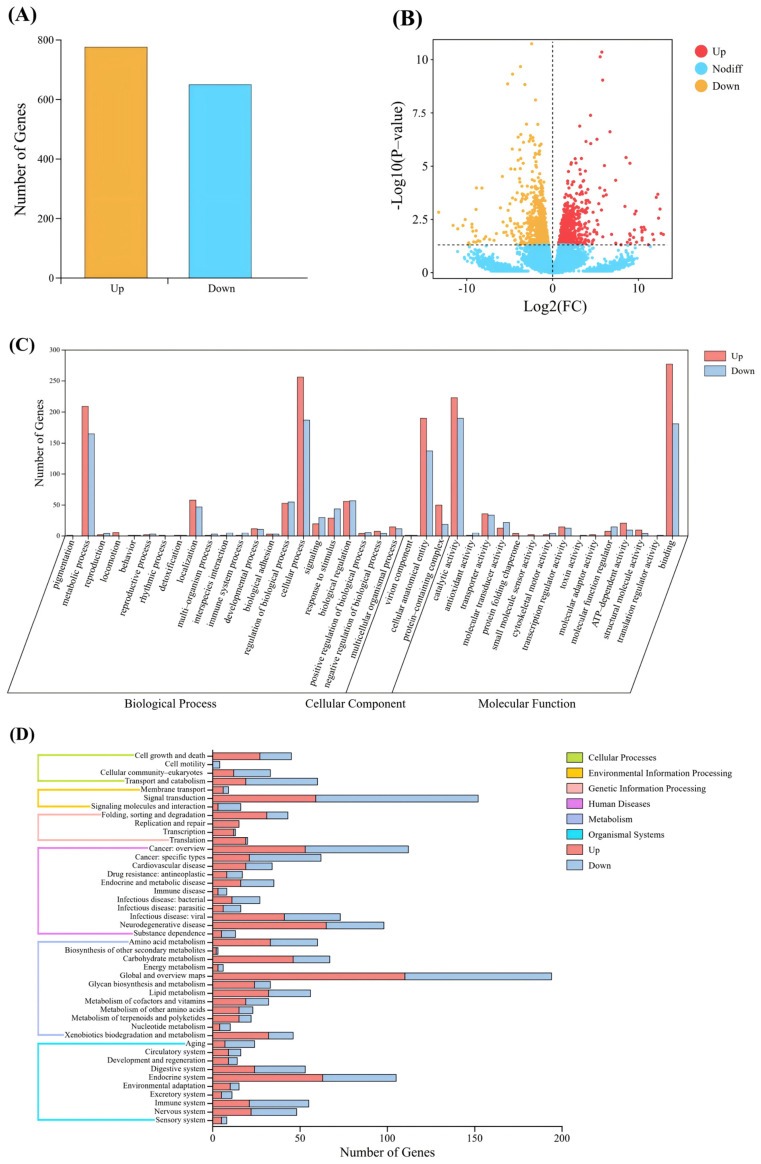
Transcriptomic profiling of DEGs in *A. glabripennis*. (**A**) Number of upregulated and downregulated genes among identified DEGs. Genes with log_2_ fold change (log_2_FC; Treatment TPM/Control TPM) > 0 were classified as upregulated, whereas those with log_2_FC < 0 were classified as downregulated. (**B**) Volcano plot illustrating the overall distribution of DEGs between treatment and control groups. (**C**) GO functional enrichment analysis of DEGs. (**D**) KEGG pathway enrichment analysis of DEGs.

A total of 1426 DEGs were identified (FDR < 0.05, |log_2_FC| > 1), including 776 up-regulated and 650 down-regulated genes in the treatment group (Figure 1A). Volcano plots analysis confirmed substantial expression changes, with several olfactory-related genes among the most highly upregulated DEGs (Figure 1B).

### 3.3. Functional Annotation of DEGs

GO enrichment analysis classified the DEGs into three main categories: Biological Process, Molecular Function, and Cellular Component. Within the Biological Process category, genes involved in metabolic processes (209 genes) and cellular processes (256 genes) were most prominently enriched. In the Molecular Function category, genes associated with binding (223 genes) and catalytic activity (277 genes) were predominant (Figure 1C). KEGG pathway analysis further categorized the DEGs into six major classes. Notably, within the Environmental Information Processing category, 152 genes were associated with signal transduction, of which 59 were upregulated—including numerous OBP-related genes (Figure 1D).

### 3.4. Identification and Bioinformatics Analysis of AglaOBPs

**Figure 2:**
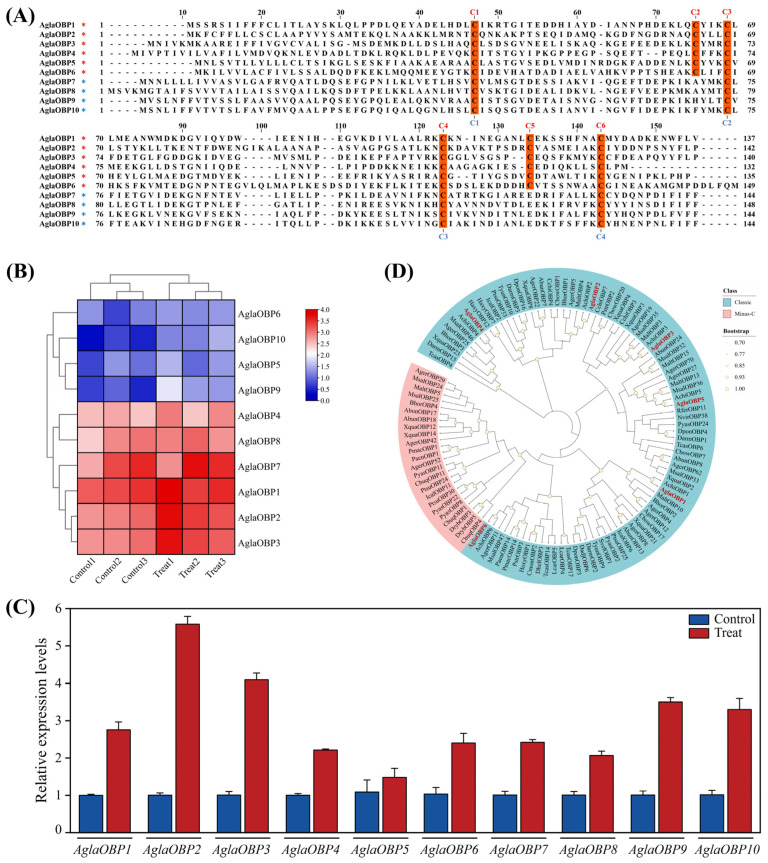
Characterization and expression analysis of OBPs in *A. glabripennis* antennae. (**A**) Multiple sequence alignment of the 10 identified OBPs; the six conserved cysteine residues are highlighted in red. (**B**) Expression profiles across six antennal transcriptome samples (Control1–3 and Treat1–3), normalized as log_10_(TPM + 1). (**C**) qRT-PCR validation of the 10 upregulated OBP genes in female antennae (Control vs. Treat). Data are presented as mean ± SE (*n* = 3). (**D**) Phylogenetic tree of Classic OBPs from *A. glabripennis* and 29 other insect species. Bootstrap values ≥ 0.7 are shown at nodes. Species abbreviations are provided in the figure (Table S4).

A total of ten OBP genes (designated *AglaOBP1*–*AglaOBP10*) were identified as significantly upregulated in the female *A. glabripennis* antennal transcriptome after exposure to *E. angustifolia* volatiles, compared with the unexposed control group. All contained complete ORFs encoding proteins of 132–149 amino acids. Sequence homology analysis showed 100% identity with OBPs from *A. glabripennis* (Table S3). Each AglaOBP contained a predicted N-terminal signal peptide. The six Classic OBPs (AglaOBP1–AglaOBP6) contained six conserved cysteines (C1-X_24–27_-C2-X_3_-C3-X_36–43_-C4-X_8–12_-C5-X_8_-C6), while the four Minus-C OBPs (AglaOBP7–AglaOBP10) contained four conserved cysteines (C1-X_30_-C2-X_37_-C3-X_19_-C4) (Figure 2A). Tissue-specific expression profiling of the transcriptome revealed that *AglaOBP1*, *AglaOBP2*, *AglaOBP3*, and *AglaOBP7* were highly enriched in antennae (Figure 2B). To validate the reliability of the transcriptome sequencing results, we performed qRT-PCR analysis on the 10 differentially expressed OBP genes using the same batch of female antenna samples. The results showed that the expression trends of all AglaOBP genes were highly consistent with the RNA-seq data (Figure 2B,C). Phylogenetic analysis of 106 OBPs from 29 other insect species clustered all AglaOBPs with *Anoplophora chinensis* OBPs, reflecting their close evolutionary relationships (Figure 2D).

### 3.5. Tissue-Specific Expression of Classic OBPs

**Figure 3:**
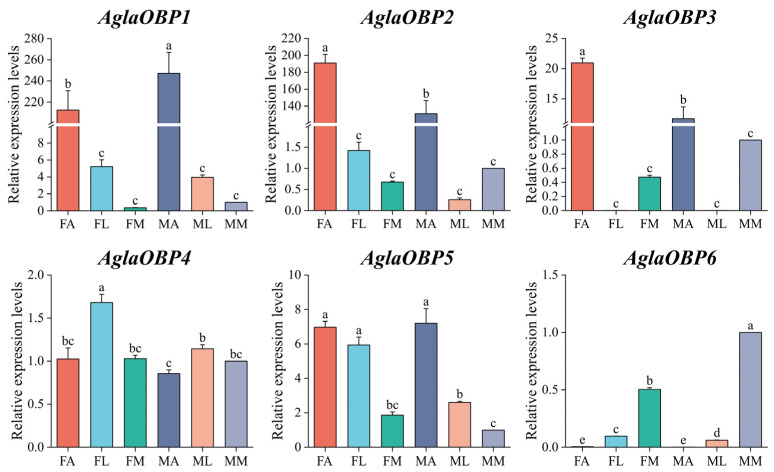
Tissue-specific expression of AglaOBPs by qRT-PCR (mean ± SE). Statistical significance was analyzed by one-way ANOVA followed by Duncan’s multiple comparison test (*p* < 0.05). Bars with different lowercase letters are significantly different, while bars with the same letter show no significant difference. FA, female antennae; MA, male antennae; FL, female legs; ML, male legs; FM, female maxillary palps; MM, male maxillary palps. Expression is normalized to MM (set to 1).

For the six Classic AglaOBP genes, qRT-PCR analysis revealed distinct tissue- and sex-specific expression patterns among the AglaOBP genes (Figure 3). *AglaOBP1*, *AglaOBP2*, and *AglaOBP3* were significantly enriched in the antennae of both sexes (*p* < 0.05), with *AglaOBP1* exhibiting significantly higher expression in male antennae than in female antennae, while *AglaOBP2* and *AglaOBP3* showed significantly higher expression in female antennae. All three genes had significantly lower expression levels in the legs and maxillary palps of both sexes compared to the antennae. *AglaOBP4* showed predominant expression in female legs, and *AglaOBP5* was highly expressed in both antennae and female legs. In contrast, *AglaOBP6* expression was largely restricted to male maxillary palps, suggesting potential non-olfactory functions for these genes beyond chemosensation.

### 3.6. Molecular Docking Results of AglaOBPs with (+)-Longifolene

Signal peptides were removed prior to homology modeling of the three major candidate OBPs—AglaOBP1, AglaOBP2, and AglaOBP3—which showed high and specific antennal expression in both sexes of *A. glabripennis*. Template searches identified OBP12 (UniProt ID: A0A1W5XGK3) and OBP3 (UniProt ID: A0A2D1LVP0) from *A. glabripennis* as optimal models for AglaOBP1 and AglaOBP3, respectively, both with 100% sequence identity. AglaOBP2 aligned best with OBP1 (UniProt ID: U6BEX5) from *Batocera horsfieldi*, exhibiting 81% identity. The selected models had high Global Model Quality Estimation (GMQE) scores of 0.97, 0.96, and 0.96 for AglaOBP1, AglaOBP2, and AglaOBP3, respectively, all exceeding the 0.96 threshold and confirming their suitability for homology modeling in SWISS-MODEL.

Stereochemical evaluation with PROCHECK revealed that 93.6%, 93.7%, and 94.8% of residues in AglaOBP1, AglaOBP2, and AglaOBP3, respectively, occupied the most favored regions of Ramachandran plots, with all remaining residues located in additionally allowed regions. ERRAT scores (89.19%, 100%, and 98.10%) and ProSA Z-scores (−6.01, −7.25, and −6.91) further confirmed structural reliability, indicating accurate stereochemistry and favorable energy profiles. Collectively, these assessments validate the models for downstream docking studies.

Molecular docking analyses with 22 *E. angustifolia* volatiles (previously identified by our group) revealed the lowest binding affinities for isobutanol (−3.5, −3.3, and −2.9 kcal·mol^−1^ for AglaOBP1, AglaOBP2, and AglaOBP3, respectively) and the highest affinities for (+)-Longifolene (−8.5, −8.1, and −6.8 kcal·mol^−1^) (Table 1). Based on these results, the interactions of AglaOBP1, AglaOBP2, and AglaOBP3 with (+)-Longifolene were selected for further detailed analysis.

**Figure 4:**
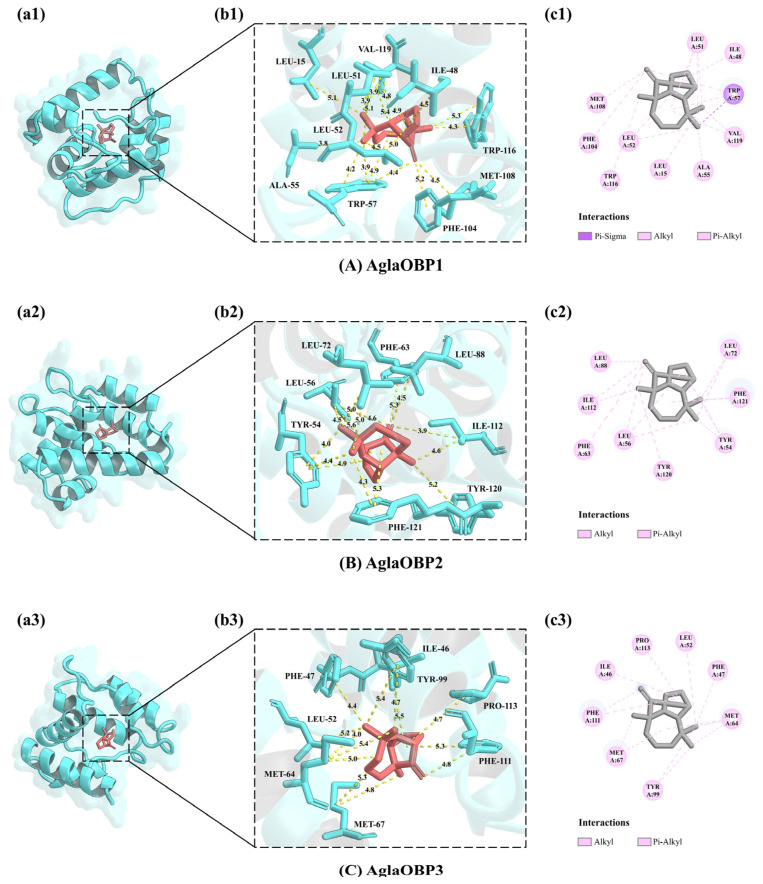
Docking interactions of AglaOBPs with (+)-Longifolene from *E. angustifolia*. (**A**–**C**) AglaOBP1–3 complexes: (**a1**–**a3**) 3D structure; (**b1**–**b3**) detailed binding mode; (**c1**–**c3**) intermolecular forces.

AglaOBP1, AglaOBP2, and AglaOBP3 each formed stable docking complexes with (+)-Longifolene, which were predominantly stabilized by hydrophobic interactions, consistent with the ligand’s hydrophobic character. Notably, distinct differences in amino acid composition and interaction patterns were observed among their respective binding pockets. The AglaOBP1 pocket is primarily composed of leucine and alanine residues, facilitating ligand binding via hydrophobic contacts. In AglaOBP2, tyrosine and phenylalanine residues dominate the pocket, where hydrophobic interactions are complemented by π–π stacking between aromatic side chains, enhancing complex stability. The AglaOBP3 binding pocket features methionine and other hydrophobic residues, with stability maintained through a combination of hydrophobic contacts and van der Waals forces (Figure 4).

### 3.7. MD Simulation

**Figure 5:**
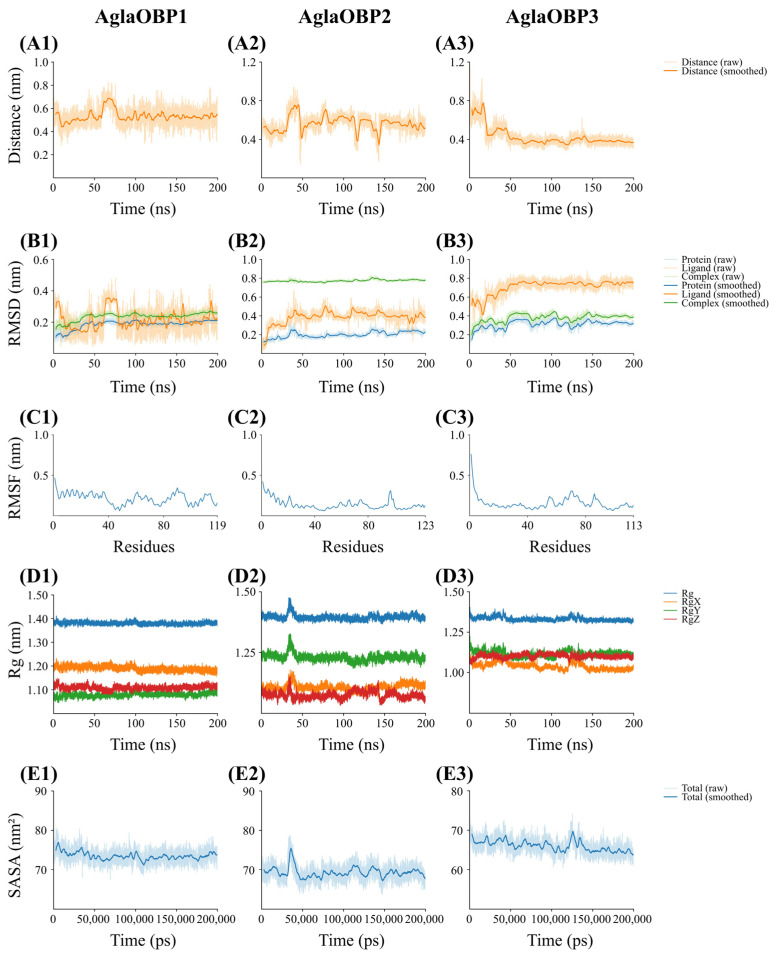
200 ns MD simulations of AglaOBP1–3 complexes with (+)-Longifolene. (**A1**–**E1**) AglaOBP1; (**A2**–**E2**) AglaOBP2; (**A3**–**E3**) AglaOBP3. (**A1**–**A3**) COM; (**B1**–**B3**) RMSD; (**C1**–**C3**) RMSF; (**D1**–**D3**) Rg; (**E1**–**E3**) SASA. Raw and smoothed data are shown as light and solid lines, respectively.

Following 200 ns MD simulations of the AglaOBP1, AglaOBP2, and AglaOBP3 complexes with (+)-Longifolene, distinct dynamic behaviors were observed. For the AglaOBP1 complex, the center-of-mass (COM) distance remained stable at 0.4–0.6 nm, reflecting steady ligand binding (Figure 5(A1)). The root-mean-square deviation (RMSD) converged to 0.2–0.3 nm after 40 ns (Figure 5(B1)), while root-mean-square fluctuation (RMSF) values for all residues remained below 0.5 nm (Figure 5(C1)). The radius of gyration (Rg) ranged from 1.3 to 1.4 nm (Figure 5(D1)), and the solvent-accessible surface area (SASA) stabilized at 70–80 nm^2^, indicating a rigid conformation with minimal structural rearrangement (Figure 5(E1)).

In the AglaOBP2 complex, the COM distance fluctuated initially, reaching up to 0.8 nm before stabilizing at 0.4–0.6 nm after 50 ns (Figure 5(A2)). The RMSD exhibited greater variation, converging to 0.7–0.8 nm, suggesting ligand-induced conformational adjustments (Figure 5(B2)). All residues presented RMSF values lower than 0.5 nm (Figure 5(C2)). The Rg transiently increased to 1.5 nm before returning to 1.3–1.4 nm (Figure 5(D2)), while SASA remained 65–75 nm^2^, indicating substantial structural stabilization upon ligand binding (Figure 5(E2)).

In contrast, the AglaOBP3 complex exhibited the fastest binding kinetics, with the COM distance decreasing sharply within 50 ns and stabilizing at 0.3–0.5 nm (Figure 5(A3)). The RMSD converged to 0.3–0.4 nm (Figure 5(B3)), and RMSF values for binding-pocket residues, although slightly higher than in other complexes, remained below 0.5 nm (Figure 5(C3)). The Rg remained confined to 1.3–1.4 nm (Figure 5(D3)), and SASA was the lowest among the three systems (60–70 nm^2^) (Figure 5(E3)). These results demonstrate that AglaOBP3 achieves superior structural compactness and dynamic stability. The final thermodynamic binding affinity was quantitatively confirmed by subsequent MM/PBSA calculations.

**Figure 6:**
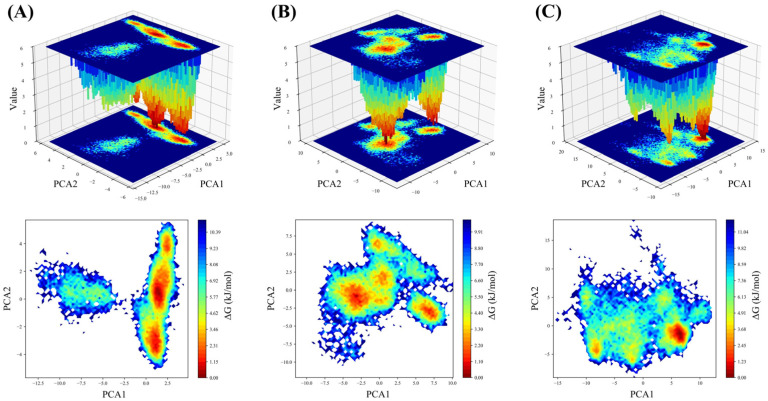
Free energy landscape of AglaOBP1–3 complexes with (+)-Longifolene from *E. angustifolia*. (**A**) AglaOBP1; (**B**) AglaOBP2; (**C**) AglaOBP3.

The free energy landscapes of all three complexes were further analyzed via principal component analysis (PCA) (Figure 6). For AglaOBP1, the conformations clustered within three deep energy minima with clear boundaries, showing the most concentrated conformational distribution, reflecting limited conformational fluctuation and high conformational stability. AglaOBP2 displayed one dominant central cluster and two smaller secondary clusters, revealing moderate conformational dispersion, indicating pronounced conformational diversity and lower stability. In contrast, AglaOBP3 exhibited two broad, diffuse low-energy regions with indistinct boundaries, with conformations spread across the entire sampled PCA space, representing the most dispersed conformational distribution.

### 3.8. MM/PBSA Analysis and Residue Decomposition

MM/PBSA binding free energy calculations (using a 10 Å radius around the ligand) further quantified the binding affinities, confirming that AglaOBP1, AglaOBP2, and AglaOBP3 all bound (+)-Longifolene strongly (Table 2). Among them, AglaOBP3 exhibited the strongest binding affinity (ΔG_bind_ = −30.94 ± 2.57 kcal·mol^−1^), surpassing AglaOBP1 (−25.55 ± 1.91 kcal·mol^−1^) and AglaOBP2 (−24.65 ± 2.76 kcal·mol^−1^). Energy decomposition revealed that ΔE_vdw_ was the primary thermodynamic driver in all complexes, with values of −28.22 ± 1.72, −29.04 ± 2.28, and −33.03 ± 2.07 kcal·mol^−1^ for AglaOBP1, AglaOBP2, and AglaOBP3, respectively. The large ΔE_vdw_ value for AglaOBP3 highlights its optimal hydrophobic complementarity with (+)-Longifolene. ΔE_ele_ were comparatively weak for AglaOBP1 (−2.71 ± 3.55 kcal·mol^−1^) and AglaOBP2 (−5.05 ± 5.21 kcal·mol^−1^), whereas AglaOBP3 displayed a substantially negative ΔE_ele_ (−24.82 ± 3.31 kcal·mol^−1^), reflecting strong charge complementarity. Solvation effects were similar across all three proteins, with positive ΔG_PB_ and negative ΔG_SA_, indicating that hydrophobic burial consistently enhanced complex stability.

**Figure 7:**
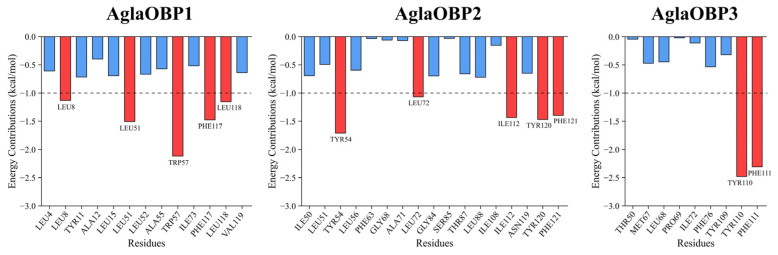
Residue-level energy contributions of AglaOBPs binding (+)-Longifolene (within 10 Å of the ligand). Negative ΔG_residue_ values represent favorable contributions to protein–ligand binding. The horizontal dashed line at −1 kcal·mol^−1^ marks the predefined cutoff for key ligand-binding residues, with red bars indicating residues meeting this criterion.

Per-residue energy decomposition identified key contributors to ligand binding (ΔG_residue_ < −1 kcal·mol^−1^) (Figure 7). In AglaOBP1, critical residues included Leu8, Leu11, Leu51, Trp57, and Phe117, which are primarily hydrophobic and aromatic, stabilizing the ligand via van der Waals interactions and hydrophobic packing. The key residues in AglaOBP2—Tyr54, Leu72, Ile112, Tyr120, and Phe121—are also predominantly hydrophobic and aromatic, with π–π stacking between aromatic side chains further enhancing ligand stabilization. For AglaOBP3, Tyr110 and Phe111 were the dominant contributors. Their combined hydrophobic packing, potential π–π interactions, and complementary polar contacts establish a more diversified and robust molecular recognition mode, consistent with the protein’s superior binding affinity and stability.

## 4. Discussion

Odorant-binding proteins (OBPs) function as key carriers of pheromones and plant volatiles in insect chemoreception [23]. In this study, transcriptomic analysis revealed that 10 OBP genes were significantly upregulated in female *A. glabripennis* antennae after 6 h exposure to *E. angustifolia* volatiles. In this study, we re-isolated exact orthologous genes whose functional annotations have already been mapped by the automatic curation of NCBI. Although this number is lower than those reported in *A. chinensis* (46 OBPs) [24], *Semanotus bifasciatus* (32 OBPs) [25], and *Glenea cantor* (29 OBPs) [26], it exceeds that identified in *B. horsfieldi* (7 OBPs) [27]. OBP diversity is thought to reflect environmental chemical complexity, enabling functional specialization [28]. By focusing on OBPs that were responsive to *E. angustifolia* volatiles, our study highlights the core components of the antennal olfactory response in *A. glabripennis*.

Sequence alignment classified four OBPs as Minus-C members, lacking C2 and C5, and six as Classic OBPs containing the conserved motif (C_1_-X_24–27_-C_2_-X_3_-C_3_-X_36–43_-C_4_-X_8–12_-C_5_-X_8_-C_6_) with six cysteine residues forming the structural core. This pattern agrees with previous reports in adult *A. glabripennis* [22], supporting evolutionary conservation within cerambycid beetles. The proteins ranged from 132 to 149 amino acids (average 142 aa), consistent with OBPs from other insects, including *Frankliniella occidentalis* [29], *Megachile saussurei* [30], and *Sirex noctilio* [31]. Such conserved length likely maintains structural stability and binding-pocket integrity, underscoring functional conservation across insect taxa.

Phylogenetic analysis indicated clear divergence among the six Classic *AglaOBPs*. As OBPs are highly sequence-divergent—even within species [32]—functional differentiation is expected. The highest homology was observed with *A. chinensis*, suggesting conserved physiological roles in congeneric species. qRT-PCR further revealed tissue- and sex-specific specialization. *AglaOBP1*, *AglaOBP2*, and *AglaOBP3* were significantly enriched in antennae compared to legs and maxillary palps, which may indicate their primary roles in olfactory perception. Notably, their sex-specific expression patterns may provide functional clues: *AglaOBP1* was significantly higher in male antennae, suggesting that it could be involved in detecting female-emitted sex pheromones or plant cues, potentially reflecting the heightened olfactory demands of mate-seeking males. Conversely, *AglaOBP2* and *AglaOBP3* were significantly upregulated in female antennae, implying that they might participate in female-specific behaviors, such as locating the *E. angustifolia* trap tree for oviposition. In contrast, *AglaOBP4*, *AglaOBP5*, and *AglaOBP6* were predominantly expressed in legs or maxillary palps, suggesting possible non-olfactory functions. Similar patterns occur in *Monochamus alternatus*, where antennal *MaltOBP10* mediates host volatile detection, while non-antennal *MaltOBP9* likely participates in gustation [33]. Based on expression profiles and known olfactory functions [34], *Agla*OBP1, *Agla*OBP2, and *AglaOBP3* likely represent core odor recognition proteins for *E. angustifolia* volatiles.

Molecular docking demonstrated that AglaOBP1, AglaOBP2, and AglaOBP3 bind to 22 *E. angustifolia* volatiles, with (+)-Longifolene showing the lowest binding energies, suggesting a likely central role in the recognition of *E. angustifolia* volatiles. This aligns with reports that (+)-Longifolene attracts *A. chinensis* [35] and other insects [36]. Moreover, our previous electrophysiological data showed strong antennal responses of female *A. glabripennis* to (+)-Longifolene [37], reinforcing its potential role as a key trap tree orientation signal.

Docking analysis also indicated that hydrophobic interactions dominate binding, with no hydrogen bonds detected. Although hydrogen bonding often contributes to OBP–ligand interactions, several OBPs rely primarily on hydrophobic and van der Waals forces [38,39]. The apparent discrepancy between dynamic parameters and free energy landscape profiles arises from divergent conformational evolution. AglaOBP1 remained stably confined to a single local energy minimum throughout the simulation, displaying minimal conformational rearrangement and stable clustered low-energy basins. AglaOBP2 showed moderate conformational fluctuation and a dispersed energy distribution, representing an intermediate conformational state between the other two proteins. In contrast, AglaOBP3 underwent conformational rearrangement and crossed the energy barrier during the early simulation phase, thereafter maintaining a stable optimal conformation at the global energy minimum. Its diffuse free energy landscape, projected from the entire simulation trajectory, captures the complete conformational transition process from the initial docking pose to the final stable bound state. All MM/PBSA binding free energy calculations were performed on the full production trajectories, and the well-converged structural parameters for all complexes ensure the reliability of the thermodynamic results. Collectively, dynamic parameters confirm that AglaOBP3 exhibits the highest structural compactness and dynamic stability toward (+)-Longifolene.

MM/PBSA and per-residue decomposition analyses identified van der Waals interactions as the principal energetic driver, consistent with other insect OBPs such as those in *Glyphodes pyloalis* [40]. Notably, *Agla*OBP3 exhibited an additional strong electrostatic contribution, accounting for its enhanced affinity and possibly reflecting adaptive charge complementarity within its binding pocket. Phenylalanine (Phe) residues emerged as shared key contributors across all three OBPs. Given their capacity for π–π stacking and strong hydrophobic interactions, Phe residues likely play a conserved structural and functional role in stabilizing OBP–ligand complexes [41,42].

## 5. Conclusions

This study elucidates the molecular basis of *E. angustifolia* volatile recognition in *A. glabripennis*. Transcriptomic analysis identified 10 OBP genes upregulated in female antennae following exposure to *E. angustifolia* volatiles, with three Classic OBPs (*AglaOBP1*–*AglaOBP3*) showing strong antennal enrichment. Integrated docking, molecular dynamics, and MM/PBSA analyses consistently highlighted (+)-Longifolene as a key ligand. Binding was primarily driven by van der Waals interactions, with conserved phenylalanine residues playing critical stabilizing roles. Among the candidate Classic OBPs, AglaOBP3 exhibited the strongest binding affinity, optimal electrostatic complementarity, and superior structural stability, supporting its putative central role in (+)-Longifolene perception. These findings uncover new molecular clues underlying the olfactory recognition of dead-end trap-tree volatiles by *A. glabripennis*, further enhance our comprehension of its olfactory processes, and establish a theoretical framework for future in vitro/in vivo functional validation of OBPs, as well as the development of semiochemical-based sustainable management strategies for this invasive wood-boring pest.

## Figures and Tables

**Table 1:** Molecular docking binding energies of AglaOBPs with 22 *E. angustifolia* volatiles.

Compound Name	CAS Number	Molecular Formula	AglaOBP1 (kcal·Mol^−1^)	AglaOBP2 (kcal·Mol^−1^)	AglaOBP3 (kcal·Mol^−1^)
Octane	111-65-9	C_8_H_18_	−4.8	−4.5	−3.9
Heptane, 2,4-dimethyl-	2213-23-2	C_9_H_20_	−5.0	−5.3	−4.3
Octane, 4-methyl-	2216-34-4	C_9_H_20_	−4.8	−5.1	−4.1
Nonane	111-84-2	C_9_H_20_	−4.7	−4.7	−4.0
Decane	124-18-5	C_10_H_22_	−4.8	−4.9	−4.2
Undecane	1120-21-4	C_11_H_24_	−5.1	−5.0	−4.2
Dodecane	112-40-3	C_12_H_26_	−5.1	−5.3	−4.0
Isobutanol	78-83-1	C_4_H_10_O	−3.5	−3.3	−2.9
p-Cymen-7-ol	536-60-7	C_10_H_14_O	−6.5	−6.4	−5.3
Furfural	98-01-1	C_5_H_4_O_2_	−4.1	−3.9	−3.5
Benzaldehyde	100-52-7	C_7_H_6_O	−4.9	−5.0	−4.1
Benzaldehyde, 4-(1 methylethyl)-	122-03-2	C_10_H_12_O	−6.3	−6.5	−5.3
3-Carene	13466-78-9	C_10_H_16_	−6.5	−6.7	−5.1
(+)-alpha-Pinene	7785-70-8	C_10_H_16_	−6.2	−6.3	−5.0
D-Limonene	5989-27-5	C_10_H_16_	−6.4	−6.6	−5.0
(+)-Longifolene	475-20-7	C_15_H_24_	−8.5	−8.1	−6.8
α-Pinene	80-56-8	C_10_H_16_	−6.1	−6.4	−5.0
Ethanone, 1-(4-ethylphenyl)-	937-30-4	C_10_H_12_O	−6.3	−6.5	−5.2
Butyl acrylate	141-32-2	C_7_H_12_O_2_	−4.5	−4.1	−3.9
o-Cymene	527-84-4	C_10_H_14_	−6.4	−6.6	−5.0
Benzene, (1,1-dimethylpropyl)-	2049-95-8	C_11_H_16_	−6.7	−6.7	−5.2
Benzene, pentyl-	538-68-1	C_11_H_16_	−5.9	−6.3	−5.2

**Table 2:** Binding Free Energy (kcal·mol^−1^) of AglaOBPs with *E. angustifolia* Volatile Compound (+)-Longifolene.

System	ΔEvdw	ΔEele	ΔGPB	ΔGSA	ΔGbind
AglaOBP1	−28.22 ± 1.72	−2.71 ± 3.55	8.88 ± 3.69	−3.49 ± 0.15	−25.55 ± 1.91
AglaOBP2	−29.04 ± 2.28	−5.05 ± 5.21	13.05 ± 5.67	−3.60 ± 0.21	−24.65 ± 2.76
AglaOBP3	−33.03 ± 2.07	−24.82 ± 3.31	30.41 ± 2.51	−3.51 ± 0.14	−30.94 ± 2.57

## Data Availability

All relevant data supporting this research are included in this article and the Supplementary File. The original reads of transcriptome sequencing from this study were uploaded to the NCBI Sequence Read Archive with accession number PRJNA1403806. The sequences of 10 AglaOBPs were also submitted to Genbank with accession numbers PZ182192–PZ182201.

## Supplementary Materials

The following supporting information can be downloaded at https://www.mdpi.com/article/10.3390/insects17070666/s1. Table S1: Gene-specific primer sequences used for qRT-PCR analysis; Table S2: Summary of transcriptome sequencing data from female *A. glabripennis* antennae; Table S3: Sequence characteristics of the 10 OBPs identified in *A. glabripennis* antennae; Table S4: Species abbreviations used in the phylogenetic tree of Classic OBPs.

## References

[1] Hansson B.S., Stensmyr M.C. (2011). Evolution of Insect Olfaction. Neuron.

[2] Vosshall L.B., Stocker R.F. (2007). Molecular Architecture of Smell and Taste in *Drosophila*. Annu. Rev. Neurosci..

[3] Brito N.F., Moreira M.F., Melo A.C.A. (2016). A look inside odorant-binding proteins in insect chemoreception. J. Insect Physiol..

[4] Pelosi P., Zhou J.-J., Ban L.P., Calvello M. (2006). Soluble proteins in insect chemical communication. Cell. Mol. Life Sci..

[5] Chen X.F., Lei Q., Liang C.H., Wang J.J., Jiang H.B. (2025). A case study on the γ-octalactone induced expression of *Obp83g-2* in *Bactrocera dorsalis* (Hendel) revealed the transcriptional regulation of insect odorant binding protein. Commun. Biol..

[6] Hekmat-Scafe D.S., Scafe C.R., McKinney A.J., Tanouye M.A. (2002). Genome-wide analysis of the odorant-binding protein gene family in *Drosophila melanogaster*. Genome Res..

[7] Gong D.P., Zhang H.J., Zhao P., Xia Q.Y., Xiang Z.H. (2009). The Odorant Binding Protein Gene Family from the Genome of Silkworm, *Bombyx mori*. BMC Genom..

[8] Wang L.X., Li C.C., Luo Y.Q., Wang G.J., Dou Z.P., Haq I.U., Cui M.M. (2023). Current and future control of the wood-boring pest *Anoplophora glabripennis*. Insect Sci..

[9] Luo L.P., Wang X.Y., Yang Z.Q., Zhao J.X., Tang Y.L. (2018). Progress in Biological Control over *Anoplophora glabripennis*. Biol. Disaster Sci..

[10] Shao P.P., Luo J.Y., Zhang R., Liu J.F., Cao D.D., Su Z., Wei J.R. (2025). Methyl Jasmonate Enhances the Resistance of *Populus alba* var. *pyramidalis* Against *Anoplophora glabripennis* (Coleoptera: Cerambycidae). Insects.

[11] Geib S.M., Tien M., Hoover K. (2010). Identification of proteins involved in lignocellulose degradation using in gel zymogram analysis combined with mass spectroscopy-based peptide analysis of gut proteins from larval Asian longhorned beetles, *Anoplophora glabripennis*. Insect Sci..

[12] Qi R.H., Pei J.H., Zhou Q., Hao K.Y., Tian Y., Ren L.L., Luo Y.Q. (2024). Comparative Metabolic Defense Responses of Three Tree Species to the Supplemental Feeding Behavior of *Anoplophora glabripennis*. Int. J. Mol. Sci..

[13] Wei J.R., Yang Z.Q., Poland T.M., Du J.W. (2009). Parasitism and olfactory responses of *Dastarcus helophoroides* (Coleoptera: Bothrideridae) to different Cerambycid hosts. BioControl.

[14] Liu H.P., Bauer L.S., Zhao T., Gao R.T., Poland T.M. (2016). Seasonal abundance and development of the Asian longhorned beetle and natural enemy prevalence in different forest types in China. Biol. Control.

[15] Li C.C., Pei J.H., Wang L.X., Tian Y., Ren L.L., Luo Y.Q. (2024). Interactions at the Oviposition Scar: Molecular and Metabolic Insights into *Elaeagnus angustifolia*’s Resistance Response to *Anoplophora glabripennis*. Int. J. Mol. Sci..

[16] Yang Z.J., Wang L.X., Ren L.L., Wang X.Q., Wang X.B., Chen Y.L. (2024). Planting model of dead-end trap tree *Elaeagnus angustifolia* and resistant host tree *Populus alba* var. *pyramidalis* for ecological self-regulation of the *Anoplophora glabripennis* disaster. Chin. For. Pest Dis..

[17] Zhao C.Y., Wang G.J., Yang Z.J., Wang X., Li W.X., Ren L.L., Wang L.X. (2025). Study on host selection of *Anoplophora glabripennis* to several tree species in the Hexi Corridor. Chin. J. Appl. Entomol..

[18] Shao P.P., Yang B.J., Su Z., Sun Z.X., Wang Z., Liu Y.T., Wei J.R. (2023). Preference of *Anoplophora glabripennis* to *Populus alba* var. *pyramidalis* and *Elaeagnus angustifolia*. For. Res..

[19] Cheng W.N., Zhang Y.D., Yu J.L., Liu W., ZhuSalzman K.Y. (2020). Functional Analysis of Odorant-Binding Proteins 12 and 17 from Wheat Blossom Midge *Sitodiplosis mosellana* Géhin (Diptera: Cecidomyiidae). Insects.

[20] Wang Z.H., Yang F., Sun A., Song J.Y., Shan S., Zhang Y.J., Wang S.N. (2022). Expressional and functional comparisons of five clustered odorant binding proteins in the brown marmorated stink bug *Halyomorpha halys*. Int. J. Biol. Macromol..

[21] Wang J.Z., Gao P., Luo Y.Q., Tao J. (2018). Characterization and expression profiling of odorant-binding proteins in *Anoplophora glabripennis* Motsch. Gene.

[22] Hu P., Wang J.Z., Cui M.M., Tao J., Luo Y.Q. (2016). Antennal transcriptome analysis of the Asian longhorned beetle *Anoplophora glabripennis*. Sci. Rep..

[23] Pelosi P., Iovinella I., Zhu J., Wang G.R., Dani F.R. (2018). Beyond chemoreception: Diverse tasks of soluble olfactory proteins in insects. Biol. Rev..

[24] Wang J.Z., Hu P., Gao P., Tao J., Luo Y.Q. (2017). Antennal transcriptome analysis and expression profiles of olfactory genes in *Anoplophora chinensis*. Sci. Rep..

[25] Li H., Hao E.H., Li Y.N., Yang H., Sun P., Lu P.F., Qiao H.L. (2022). Antennal transcriptome analysis of olfactory genes and tissue expression profiling of odorant binding proteins in *Semanotus bifasciatus* (Cerambycidae: Coleoptera). BMC Genom..

[26] Wu G.X., Su R.R., Ouyang H.L., Zheng X.L., Lu W., Wang X.Y. (2022). Antennal Transcriptome Analysis and Identification of Olfactory Genes in *Glenea cantor* Fabricius (Cerambycidae: Lamiinae). Insects.

[27] Yang H., Cai Y., Zhuo Z.H., Yang W., Yang C.P., Zhang J., Guan F.R. (2019). Transcriptome analysis in different developmental stages of *Batocera horsfieldi* (Coleoptera: Cerambycidae) and comparison of candidate olfactory genes. PLoS ONE.

[28] Sánchez-Gracia A., Vieira F.G., Rozas J. (2009). Molecular evolution of the major chemosensory gene families in insects. Heredity.

[29] Cao J.Y., Wang X., Wang S.Y., Wang Y.H., Li X.M., Wang Y.G., Wang J. (2025). Molecular Characterization and Binding Properties of Odorant Binding Protein 7 in Western Flower Thrips, *Frankliniella occidentalis* (Thysanoptera: Thripidae). J. Agric. Food Chem..

[30] Li W.Z., Kang W.J., Zhou J.J., Shang S.Q., Shi S.L. (2023). The antennal transcriptome analysis and characterizations of odorant-binding proteins in *Megachile saussurei* (Hymenoptera, Megachilidae). BMC Genom..

[31] Hao R., Li Y.N., Hao E.H., Yuan X.H., Lu P.F., Qiao H.L. (2022). Interaction Analysis of Odorant-Binding Protein 12 from *Sirex noctilio* and Volatiles from Host Plants and Symbiotic Fungi Based on Molecule Dynamics Simulation. Agronomy.

[32] Pelosi P., Iovinella I., Felicioli A., Dani F.R. (2014). Soluble proteins of chemical communication: An overview across arthropods. Front. Physiol..

[33] Li D.Z., Huang X.F., Yang R.N., Chen J.Y., Wang M.Q. (2020). Functional Analysis of Two Odorant-Binding Proteins, MaltOBP9 and MaltOBP10, in *Monochamus alternatus* Hope. Front. Physiol..

[34] Yang R.S., Zhou J.N., Hao J.X., Zhang T.T., Jiang Y.R., Liu W., Wang Y. (2025). Olfactory binding proteins: A review across the Insecta. Front. Zool..

[35] Yang H., Jiang L.J., Bao X.C., Liu H.L., Xu Q.L., Yao X.L., Li J. (2025). CeJAZ3 suppresses longifolene accumulation in *Casuarina equisetifolia*, affecting the host preference of *Anoplophora chinensis*. Pest Manag. Sci..

[36] Tang D., Chen J.N., Zhang Y.B., Tang X.Y., Wang X.M., Yu C.N., Fang W.G. (2025). Engineered Metarhizium fungi produce longifolene to attract and kill mosquitoes. Nat. Microbiol..

[37] Wang G.J. (2024). EAG Response of *Anoplophora glabripennis* to Volatiles of *Fusarium solani* and Its Infected *Elaeagnus angustifolia*. Master’s Thesis.

[38] Lartigue A., Gruez A., Spinelli S., Rivière S., Brossut R., Tegoni M., Cambillau C. (2003). The crystal structure of a cockroach pheromone-binding protein suggests a new ligand binding and release mechanism. J. Biol. Chem..

[39] Northey T., Venthur H., De Biasio F., Chauviac F.-X., Cole A., Ribeiro K.A.L., Zhou J.-J. (2016). Crystal Structures and Binding Dynamics of Odorant-Binding Protein 3 from two aphid species *Megoura viciae* and *Nasonovia ribisnigri*. Sci. Rep..

[40] Li Y.J.C., Song W.M., Wang S.S., Miao W.L., Liu Z.X., Wu F., Sheng S. (2024). Binding characteristics and structural dynamics of two general odorant-binding proteins with plant volatiles in the olfactory recognition of *Glyphodes pyloalis*. Insect Biochem. Mol. Ecol..

[41] Tian Z., Li Y., Zhou T., Ye X., Li R.C., Liu J.Y. (2020). Structure dynamics reveal key residues essential for the sense of 1-dodecanol by *Cydia pomonella* pheromone binding protein 2 (CpomPBP2). Pest Manag. Sci..

[42] Zhang Y.N., Zhang X.C., Zhu R., Yao W.C., Xu J.W., Wang M., Wu X.M. (2021). Computational and Experimental Approaches to Decipher the Binding Mechanism of General Odorant-Binding Protein 2 from *Athetis lepigone* to Chlorpyrifos and Phoxim. J. Agric. Food Chem..

