# Supplementary material for: Exploring the Olfactory Recognition of Elaeagnus angustifolia Volatiles in Anoplophora glabripennis Through Antennal Transcriptome Analysis and Molecular Characterization of Classic OBPs
Source: Insects. 2026 Jun 25;17(7):666. doi: 10.3390/insects17070666 (PMC13411824; doi:10.3390/insects17070666)
Supplement: Supplementary file 1 [file insects-17-00666-s001.zip › Supplementary Files/Table S1.docx]

Table S1. Gene-specific primer sequences used for qRT-PCR analysis.

| **Gene name** | **Forward primer (5’-3’)** | **Reverse primer (5’-3’)** |
| --- | --- | --- |
| *AglaOBP1* | TAAGGACATCGTACTCGCCG | CGTCATACATGCAGGCGTTG |
| *AglaOBP2* | AGAAGCTCATGCGGAACACT | GCACTGAGCATTTCTGTCACC |
| *AglaOBP3* | TTGTGGGTGTGTGTGTAGCA | CGTTTACTCCCGAGTCGGAT |
| *AglaOBP4* | AACAGACAAGCTTCGCCAGA | GCATAGCTGCTCGGGAGTAA |
| *AglaOBP5* | TGTCTCACCTCGATTAAGGGTC | TCAGCAAACTTGCCGTCTCT |
| *AglaOBP6* | AGCGCACTGGACCAAGATTT | TCAGCAATGTCAGCGTCAGT |
| *β-actin* | ACATCAAGGAGAAACTCTGCTACG | CTTCATGATGGAGTTGTAGGTGGT |
