# Supplementary material for: Exploring the Olfactory Recognition of Elaeagnus angustifolia Volatiles in Anoplophora glabripennis Through Antennal Transcriptome Analysis and Molecular Characterization of Classic OBPs
Source: Insects. 2026 Jun 25;17(7):666. doi: 10.3390/insects17070666 (PMC13411824; doi:10.3390/insects17070666)
Supplement: Supplementary file 1 [file insects-17-00666-s001.zip › Supplementary Files/Table S2.docx]

Table S2. Summary of transcriptome sequencing data from female A. glabripennis antennae.

| **Sample** | **Raw reads** | **Clean reads** | **Q20 (%)** | **Q30 (%)** | **Adapter (%)** | **GC (%)** |
| --- | --- | --- | --- | --- | --- | --- |
| Control1 | 45,536,632 | 45,093,200 | 99.36 | 96.99 | 0.05 | 42.63 |
| Control2 | 55,748,808 | 55,232,374 | 99.34 | 96.95 | 0.05 | 41.19 |
| Control3 | 54,775,038 | 54,211,340 | 99.22 | 96.43 | 0.05 | 43.53 |
| Treat1 | 44,432,470 | 44,036,464 | 99.41 | 97.23 | 0.05 | 39.13 |
| Treat2 | 40,841,416 | 40,468,268 | 99.12 | 95.73 | 0.05 | 43.77 |
| Treat3 | 58,434,996 | 57,866,618 | 99.39 | 97.12 | 0.04 | 40.69 |
