# Supplementary material for: Exploring the Olfactory Recognition of Elaeagnus angustifolia Volatiles in Anoplophora glabripennis Through Antennal Transcriptome Analysis and Molecular Characterization of Classic OBPs
Source: Insects. 2026 Jun 25;17(7):666. doi: 10.3390/insects17070666 (PMC13411824; doi:10.3390/insects17070666)
Supplement: Supplementary file 1 [file insects-17-00666-s001.zip › Supplementary Files/Table S3.docx]

Table S3. Sequence characteristics of the 10 OBPs identified in A. glabripennis antennae.

| **Number** | **Accession No.** | **ORF Length**  **(aa)** | **Signal peptide**  **AA** | **Best BLASTX match** | | | |
| --- | --- | --- | --- | --- | --- | --- | --- |
|  |  |  |  | **Name** | **Acc. number** | **E-value** | **Identity**  **(%)** |
| *AglaOBP1* | PZ182192 | 137 | 1-18 | general odorant-binding protein 83a-like | XP_023310142.1 | 1e-95 | 100% |
| *AglaOBP2* | PZ182193 | 142 | 1-19 | general odorant-binding protein 72-like | XP_018568151.1 | 1e-98 | 100% |
| *AglaOBP3* | PZ182194 | 140 | 1-27 | general odorant-binding protein 83a-like isoform X1 | XP_018576270.1 | 2e-95 | 100% |
| *AglaOBP4* | PZ182195 | 132 | 1-20 | uncharacterized protein LOC108904898 isoform X1 | XP_018563128.1 | 1e-81 | 100% |
| *AglaOBP5* | PZ182196 | 135 | 1-19 | general odorant-binding protein 69a-like isoform X2 | XP_018579768.1 | 7e-72 | 100% |
| *AglaOBP6* | PZ182197 | 149 | 1-16 | general odorant-binding protein 19d | XP_018563763.1 | 5e-105 | 100% |
| *AglaOBP7* | PZ182198 | 144 | 1-21 | general odorant-binding protein 83a-like | XP_018563380.1 | 3e-91 | 100% |
| *AglaOBP8* | PZ182199 | 148 | 1-25 | general odorant-binding protein 83a-like | XP_018563378.1 | 5e-102 | 100% |
| *AglaOBP9* | PZ182200 | 144 | 1-21 | general odorant-binding protein 72-like | XP_018563348.1 | 5e-99 | 100% |
| *AglaOBP10* | PZ182201 | 144 | 1-21 | general odorant-binding protein 83a-like | XP_018563349.1 | 5e-99 | 100% |
