# Supplementary material for: Exploring the Olfactory Recognition of Elaeagnus angustifolia Volatiles in Anoplophora glabripennis Through Antennal Transcriptome Analysis and Molecular Characterization of Classic OBPs
Source: Insects. 2026 Jun 25;17(7):666. doi: 10.3390/insects17070666 (PMC13411824; doi:10.3390/insects17070666)
Supplement: Supplementary file 1 [file insects-17-00666-s001.zip › Supplementary Files/Table S4.docx]

Table S4. Species abbreviations used in the phylogenetic tree of Classic OBPs.

| **Abbreviation** | **Scientific name** |
| --- | --- |
| Abun | Aromia bungii |
| Achi | Anoplophora chinensis |
| Ager | Apriona germarii |
| Bhor | Batocera horsfieldi |
| Cbow | Colaphellus bowringi |
| Cbuq | Cyrtotrachelus buqueti |
| Cchi | Callosobruchus chinensis |
| Cmon | Cryptolaemus montrouzieri |
| Dadj | Dendroctonus adjunctus |
| Darm | Dendroctonus armandi |
| Dhel | Dastarcus helophoroides |
| Dryb | Diorhabda rybakowi |
| Dpon | Dendroctonus ponderosae |
| Haxy | Harmonia axyridis |
| Ical | Ips calligraphus |
| Lcar | Lytta caraganae |
| Malt | Monochamus alternatus |
| Msal | Monochamus saltuarius |
| Nvir | Nezara viridula |
| Paen | Pyrrhalta aenescens |
| Pmac | Pyrrhalta maculicollis |
| Pstr | Phyllotreta striolata |
| Ptsu | Pagiophloeus tsushimanus |
| Pyas | Pachyrhinus yasumatsui |
| Rfer | Rhynchophorus ferrugineus |
| Svel | Sympiezomias velatus |
| Tcas | Tribolium castaneum |
| Tyun | Tomicus yunnanensis |
| Xqua | Xylotrechus quadripes |
